# Theoretical investigation of Banert cascade reaction

**DOI:** 10.1098/rsos.171075

**Published:** 2018-04-04

**Authors:** S. Bhattacharyya, K. Hatua

**Affiliations:** 1 Chemistry of Interfaces, Luleå University of Technology, Luleå 97187, Sweden; 2 Department of Chemistry, IIEST, Shibpur 711103, India

**Keywords:** triazole, Banert cascade, propargyl chloride, density functional theory, propargyl azide

## Abstract

Computational inside of Banert cascade reaction for triazole formation is studied with B3LYP/6-31G(d,p) level of theory. The reaction proceeds mainly by SN2 initial chloride displacement rather than SN2′-type attack. Furthermore, according to the rate of reaction calculation, SN2 displacement is much faster than SN2′ displacement in the order of 8. The [3,3]-sigmatropic rearrangement for the conversion of propargyl azide into triazafulvene has been proved as the rate-determining step having highest activation energy parameter. Solvent effect on total course of reaction has been found negligible. Furthermore, effects of different density functional theory functionals and functional groups on activation energies of [3,3]-sigmatropic rearrangement of propargyl azide were also studied. BHHLYP, *ω*B97XD, M062X and BMK calculated Δ*G*^‡^ are consistent with B3LYP.

## 1. Introduction

**Scheme 1.**
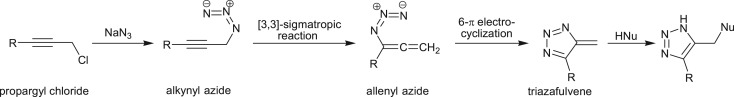
Banert cascade reaction.

Cascade reactions represent some of the most significant reactions in organic chemistry for the construction of many heterocycles, natural products, intermediates and drugs [1–3]. The application of cascade reactions in current organic chemistry can impart atom economy, minimizing chemical waste generation, step economy, as well as economy of labour and resource management. Owing to this undeniable benefit of cascade reactions, currently cascade reactions can be categorized under the banner of green chemistry. Banert cascade is one of the examples of cascade reactions for preparing 1,2,3-triazole heterocycles [4–7]. Azoles are one of the key nitrogen-based heterocyclic compounds. Among them, triazole ring is one of the important heterocyclic azole-based building blocks found in antiviral, anti-tuberculosis, antibacterial, anti-HIV and antifungal drugs [8–14]. Thus, heterocyclic molecules based on triazole represent the cornerstone of medicinal chemistry [15–18]. The first benzylic-substituted NH-triazole via [3,3]-sigmatropic rearrangement was reported by Klaus Banert in 1989. The reaction proceeds (scheme 1) by the reaction of propargyl chloride with sodium azide to give alkynyl azide, which gets easily converted into short-lived allenyl azide via [3,3]-sigmatropic rearrangement.

This short-lived allenyl azide gets cyclized via 6-π electrocyclization reaction and converts into triazafulvene intermediate. Owing to its very high dipole moment value, triazafulvene can be easily trapped by external nucleophile or excess of azides to give 1,2,3-triazole-containing benzylic system. Participation of triazafulvene as an intermediate is unique but was not detected directly yet although mechanistic evidence depicts sturdily cascade sequence as in scheme 1. However, the formation of transient triazafulvene intermediate is an unprecedented characteristic of this reaction in contrast to the conventional Cu catalysed azide–alkyne cycloaddition reactions [19–22]. Furthermore, Banert cascade reaction represents a novel tool for the selective and efficient introduction of benzylic 1,2,3-triazole system in the representative molecular architecture [23–26]. Despite the possible widespread synthetic applications of such an important reaction for the synthesis of triazoles, Banert cascade reaction has received less attention among organic chemists [27].

To the best of our knowledge, no attempts have been made to explore with atomistic insight this reaction mechanism. Thus, understanding and analysing the knowledge about the mechanism and reactivity issues could be helpful for further expanding the scope of this reaction. So far, no computational studies have been carried out on this reaction. Therefore, our main focus was to study the theoretical mechanistic insights involved in the Banert cascade reaction.

## 2. Computational details

All the reactants, products, intermediates (IM) and transition states (TS) have been optimized by Becke's three parameters exchange in conjunction with LYP (Lee, Young and Parr) correlational functional [28,29] employing a split valence double zeta quality basis accompanying ‘d’- and ‘p’-type polarization function, i.e. 6-31G(d,p) basis set has been used throughout this work. Optimized geometry of the reactants, products and intermediates has been by confirmed as a real stationary point having all real frequency obtained by vibrational analysis under harmonic approximation and the transition states are characterized by the presence of only one imaginary frequency. Intrinsic reaction coordinate (IRC) [30–32] has been carried out to confirm the optimized TS for its connectivity with corresponding intermediates in the respective potential energy hypersurface. All the calculated TS are genuine saddle points as they are connected with respective intermediates. Gas phase optimized geometry has been considered for solvation effect. Six different solvents (acetone, water, methanol, ethanol, dimethyl sulfoxide (DMSO) and acetonitrile) have been considered using the CPCM solvation model [33–36]. In continuation of our previous studies [37] of different density functional theory (DFT) functionals in predicting activation energy parameters of organic reaction mechanisms, here we also considered some popular DFT exchange-correlational functionals particularly BHHLYP [28,38], *ω*B97XD [39], M062X [40], BMK [41] and B2PLYP [42] for their relative performances. Apart from the wide acceptability of B3LYP, current DFT functional has been already proved useful and gained acceptance in recent years, for single-point calculation of the reactants, intermediates, transition states and products. However, the solvation effect has been considered only in the B3LYP method. All the thermochemical parameters are calculated at 298 K and 1 atm pressure. All the calculations have been carried out as implemented in G09 quantum chemistry package [43].

## 3. Results and discussion

**Figure 1.**
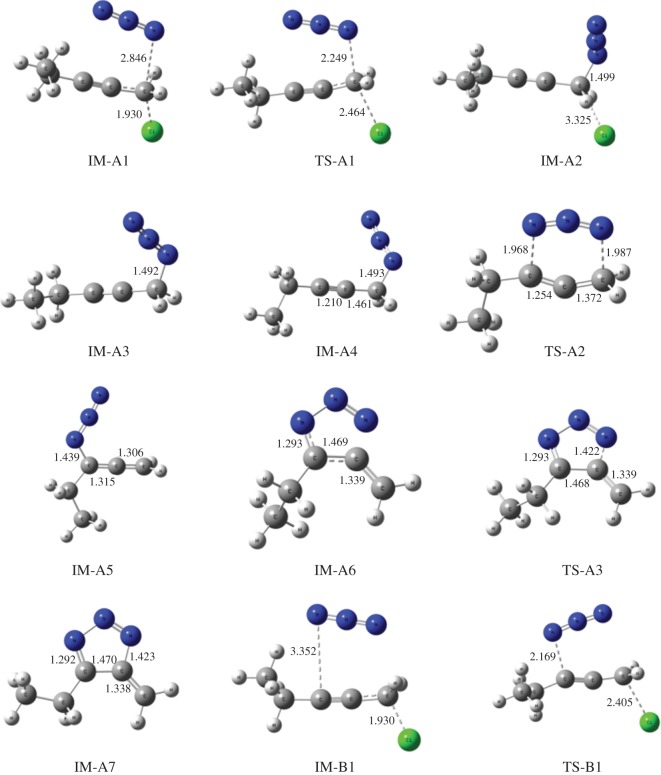
Optimized structures of intermediates and transition states with embedded bond lengths in Å.

All the optimized structures of reactants, products, intermediates and transition states are given in figure 1 with embedded bond lengths for clarity. Calculated Gibbs free energy of activation (Δ*G*^‡^) and enthalpy of activation (Δ*H*^‡^) of four transition states in gas phase as well as in different solvents are presented in table 1 for comparison, while the relative Gibbs free energy (Δ*G*) and enthalpy (Δ*H*) of different intermediates and transition states are presented in electronic supplementary material, table S1.

**Table 1.** B3LYP/6-31G(d,p) calculated Gibbs free energy of activation (Δ*G*^‡^) and enthalpy of activation (Δ*H*^‡^) of different transition states.

	gas		acetone		ethanol		methanol		acetonitrile		DMSO		water	
	Δ*G*^‡^	Δ*H*^‡^	Δ*G*^‡^	Δ*H*^‡^	Δ*G*^‡^	Δ*H*^‡^	Δ*G*^‡^	Δ*H*^‡^	Δ*G*^‡^	Δ*H*^‡^	Δ*G*^‡^	Δ*H*^‡^	Δ*G*^‡^	Δ*H*^‡^
TS-A1	3.94	2.79	3.50	2.58	4.81	2.58	6.87	2.58	3.48	2.57	3.46	2.58	3.46	2.57
TS-A2	24.04	21.30	13.20	21.20	18.47	21.19	24.10	21.19	24.11	25.42	24.11	21.19	24.11	21.20
TS-A3	1.88	0.44	1.73	0.30	1.73	0.89	1.73	0.30	1.73	0.30	1.73	0.31	1.73	0.30
TS-B1	5.10	4.48	6.75	5.58	6.23	5.06	7.28	5.52	6.76	5.60	6.77	5.61	6.78	5.62

**Figure 2.**
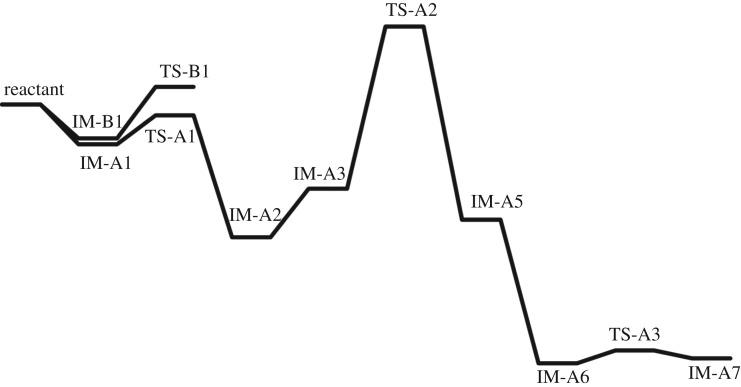
Schematic presentation of reaction profile diagram of Banert cascade reaction (in gas phase).

**Figure 3.**
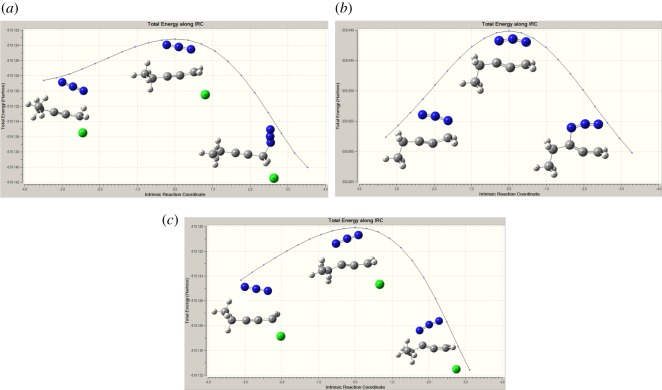
Intrinsic reaction coordinates of major transition states in the Banert cascade reaction.

**Scheme 2.**
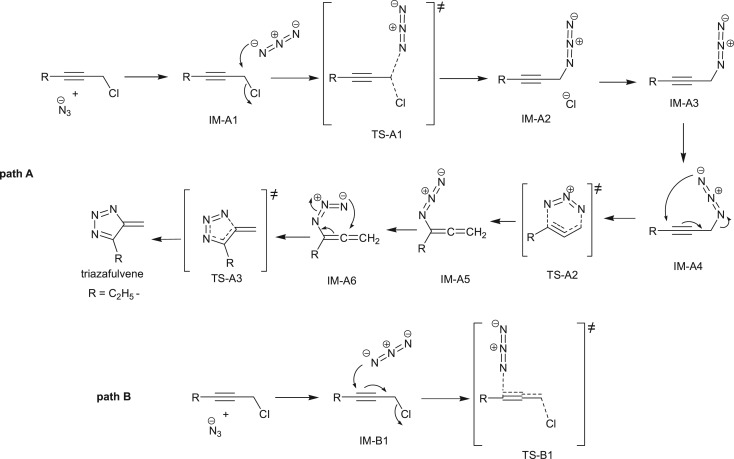
Paths A and B.

According to path A (figure 2 and scheme 2), the reaction proceeds through an azide (N3) anion attack to the ground state propargyl chloride that requires a weakly bound intermediate IM-A1 having C–N and C–Cl distances of 2.84 Å and 1.93 Å, respectively. This intermediate IM-A1 has been found stable by −5.55 kcal mol^−1^, which has undergone a rapid SN2 displacement of chloride ion through the TS-A1 transition state having significant shortening of C–N bond (2.25 Å) with concomitant elongated C–Cl distance (2.46 Å). IRC of the TS concerned is shown in figure 3*a*, which justifies the connectivity with IM-A1 and IM-A2.

**Scheme 3.**
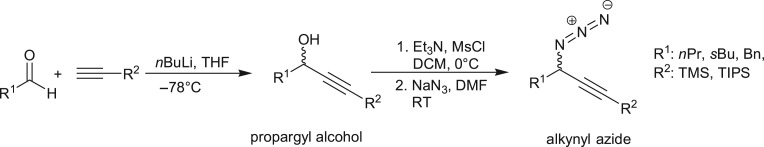
Preparation of propargyl azide from aldehydes and alkynes [45].

The intermediate IM-A2 further rearranges to propargyl azide IM-A3. This propargyl azide is the key intermediate for the Banert cascade reaction [44]. Montagnat *et al.* [45] also synthesized and isolated structurally diverse propargyl azides from aldehydes and alkynes, which also support existence of this key intermediate in Banert cascade reaction (scheme 3). Banert *et al.* also isolated various propargyl azides by previously reported methods [7,46]. Gas phase calculated activation energy barrier (Δ*G*^‡^) of TS-A1 is 3.94 kcal mol^−1^ and the rate of reaction is 8.0 × 10^9^ s^−1^. It is interesting to note that Δ*G*^‡^ of this SN2 displacement is little affected upon inclusion of solvation. Except in ethanol and methanol calculated Δ*G*^‡^ in the rest of the solvents are less by approximately 0.50 kcal mol^−1^. Although experimentally done, in most of the cases, S_N_2 displacement by N-atom in protic solvents was always more sluggish than in polar aprotic solvents [47]. In the case of Banert cascade reaction, solubility of the reactants also plays a big role for the reaction as both the reactants sodium azide and propargyl halide are opposite in nature. For example, Loren & Sharpless use the mixture of solvents (dioxane : water, 3 : 1) as well as a buffering agent NH_4_Cl to adjust the compatibility of both reactants [27].

On the other hand, according to path B, chloride ion might be displaced by SN2 attack at γ carbon atom of C≡C triple bond. This alternative synchronized SN2 displacement passes through the TS-B1 transition state having C–N and C–Cl distances of 2.17 Å and 2.40 Å, respectively. Corresponding IRC, which connects the two intermediates IM-B1 and IM-B3 along the normal coordinates, is shown in figure 3*c*. However, it should be noted that only path A has been considered as the major route for chloride displacement. There are several mechanistic evidences in favour of [3,3] sigmatropic rearrangement and concerted cycloaddition between alkyne and azide has been ruled out. From table 1, it is observed that gas phase calculated activation energy Δ*G*^‡^ of path B is quite large and also the rate of reaction slower (calculated rate of reaction for TS-B1 is 1.1 × 10^9^ s^−1^) compared to path A and upon solvation it increases significantly. Thus, chloride displacement by SN2′ might be a thermodynamically unfavourable process which supports the previous conclusion. It could be easily understood that α carbon atom bearing the Cl atom in propargyl chloride is positively polarized that invites a negatively charged azide anion (–N3) attack as a more suitable choice than the attack at negatively polarized γ carbon atom of C≡C bond.

The resulting intermediate IM-A3, after Cl displacement, was rapidly rearranged to IM-A4 for [3,3] sigmatropic shift of the attached N3 unit through the transition state TS-A2. This is the rate-determining step of Banert cascade reaction, where simultaneous C–N bond breaking and bond formation take place. IRC of corresponding [3,3] sigmatropic shift, which connects IM-A4 and TS-A2, is shown in figure 3*b*. Calculated activation energy barrier (Δ*G*^‡^) is 24.04 kcal mol^−1^ in gas phase and calculated rate of reaction is 1.47 × 10^−5^ s^−1^. It is interesting to note that Δ*G*^‡^ of this rate-determining step is not so affected upon solvation due to mutual solvation of both IM-A4 and TS-A2. The allenyl azide intermediate IM-A5 is very unstable and instantaneously cyclizes very fast via 6π-electrocyclization reaction to IM-A7 through the transition state TS-A3. Calculated Δ*G*^‡^ has been found very small (1.88 kcal mol^−1^, calculated rate of reaction is 2.6 × 10^11^ s^−1^) and it decreases significantly upon solvation. So, formed triazafulvene rapidly reacts with external nucleophile and is converted into more stable 1,2,3-NH triazole. The intermediate allenyl azide is not directly observed due to its facile cyclization reaction. However, Fotsing & Banert [44] isolated stable sulfone-based allenyl azide with 89% yield from alkynyl azide. Banert & Hagedorn also isolated allenyl azide as an intermediate by preparative gas chromatography. The stability of allenyl azides solely depends on the structure. The lower congeners are stable for a short period at room temperature [7]. Furthermore, the intermediate triazafulvene (IM-A7) may not be directly detected but may be trapped by nucleophilic solvents. Banert & Hagedorn [7] were able to detect indirectly triazafulvene intermediate in this cascade reaction using CD_3_OD as solvent which results CD_3_O-incorporated 1,2,3-ND benzyl triazole.

**Scheme 4.**
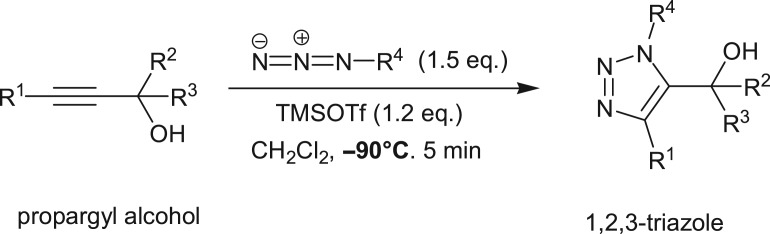
Conversion of propargylic alcohol to 1,2,3-triazole [25].

Experimentally, it is also found that propargylic systems are highly reactive which drives simultaneous conversion into products even at very low temperature (scheme 4) [25].

Although it has been shown that functionalized 1,2,3-NH triazole could be formed with a variety of chemical substituents, we consider the IM-A4 intermediate a model compound for theoretical study to fathom the effect on activation parameters (Δ*G*^‡^ and Δ*H*^‡^) by different chemical substitution (X). It is well known that electron-donating substituent facilitates the azide migration when attached to the adjacent position of the azido group, while electron-withdrawing substituent retards it. This observation can also be made experimentally.

**Scheme 5.**
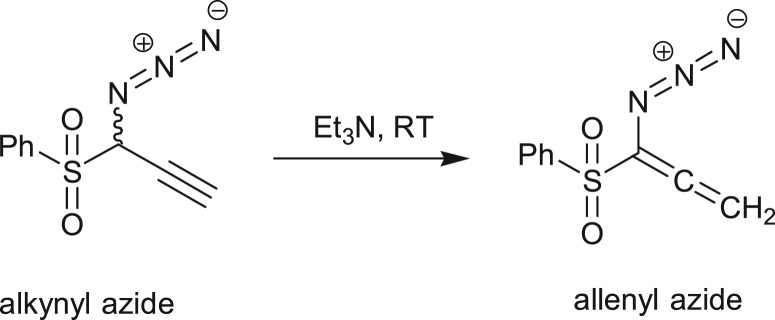
Preparation of allenyl azide [44].

The alkynyl azide containing electron-withdrawing sulfone group is stable enough to isolate at room temperature (scheme 5) [44]. A series of IM-A4 intermediates with different X (–Cl, –OH, –OMe, –NH_2_, –NMe_2_, –NO_2_, –COOH) are considered and corresponding activation energy parameters (Δ*G*^‡^ and Δ*H*^‡^) of azide migration are presented in table 2.

**Table 2.** B3LYP/6-31G(d,p) calculated Gibbs free energy of activation (Δ*G*^‡^) and enthalpy of activation (Δ*H*^‡^) for different chemical substituents in [3,3] sigmatropic reaction.

	B3LYP		BHHLYP		*ω*B97XD		M062X		BMK		B2PLYP	
	Δ*G*^‡^	Δ*H*^‡^	Δ*G*^‡^	Δ*H*^‡^	Δ*G*^‡^	Δ*H*^‡^	Δ*G*^‡^	Δ*H*^‡^	Δ*G*^‡^	Δ*H*^‡^	Δ*G*^‡^	Δ*H*^‡^
–H	24.04	21.30	29.38	28.32	26.07	24.73	25.60	24.04	23.63	25.31	21.34	21.03
–Cl	25.78	23.85	32.09	31.20	28.45	27.18	27.89	26.61	25.74	28.61	23.33	23.11
–OH	19.90	17.28	26.28	25.47	22.93	21.59	23.43	21.78	22.32	22.06	17.36	17.18
–OMe	18.53	16.55	25.32	24.66	21.73	20.86	22.34	21.21	20.77	22.33	16.14	16.54
–NH_2_	14.83	12.60	21.23	20.57	19.21	17.87	19.77	18.51	18.35	17.84	11.69	13.13
–NMe_2_	10.14	9.15	17.58	17.35	14.88	15.72	16.68	16.47	15.51	14.36	10.15	9.13
–COOH	21.07	19.25	27.85	27.07	24.74	23.91	25.05	23.84	24.63	26.61	18.09	19.83
–NO_2_	27.08	24.56	33.10	32.24	29.11	28.22	29.15	28.11	28.05	28.16	24.28	24.18

**Figure 4.**
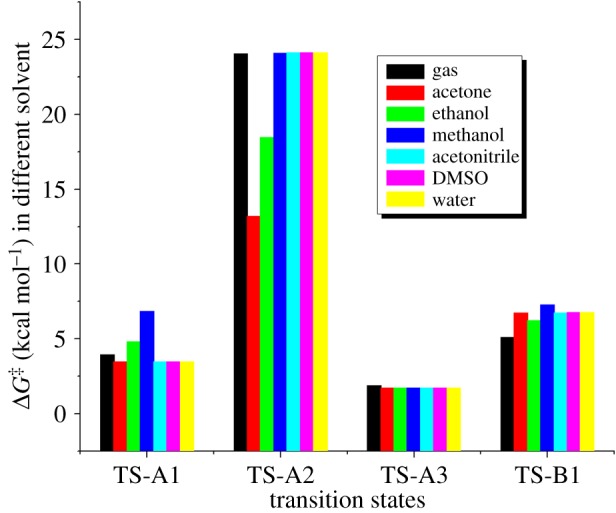
Effect of solvation of different transition states of Banert cascade reaction.

**Figure 5.**
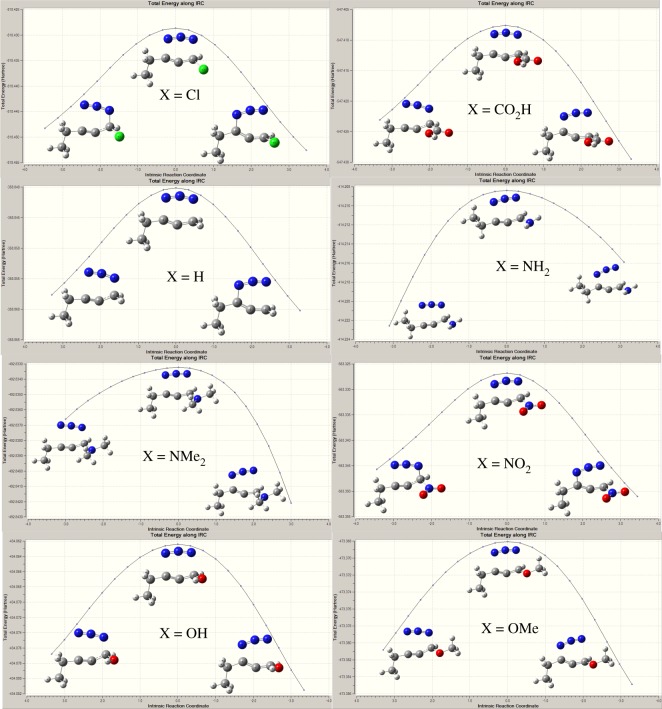
IRCs of different substitution of [3,3] sigmatropic shift reaction of Banert cascade reaction.

As solvation does not affect this migration step appreciably (figure 4), all computation has been restricted in the gas phase only. Although Loren and Sharpless reported low yield in dimethylformamide (DMF), acetone with other side products whereas in DMSO reaction halted at propargyl azide and did not proceed even after heating. In water, polymeric products are formed as propargyl derivatives are insoluble in water and can be considered neat under this condition but were able to isolate products with high yield in dioxane : water, 3 : 1 ratio [27]. Strawinska & Sas [23] isolated various types of triazolo-nucleosides via Banert cascade reaction by following two different protocols. Surprisingly, in the two-step process, successive isolation of propargyl azide in DMF and rearrangement reaction in water gave higher yield compared with one-pot protocol in dioxane–water reported by Loren & Sharpless [27]. This is possibly the solubility effects of the reactants which are not incorporated in these calculations. IRCs of all the transition states are shown in figure 5 with embedded optimized TS.

It could be easily seen that electron-donating substituent (–OH, –OMe, –NH_2_, –NMe_2_) significantly reduces the activation energy barrier, while electron-withdrawing substituent (–COOH, –NO_2_) does not affect the TS appreciably. Lowest Δ*G*^‡^ has been predicted for strongest donor –NMe_2_, which is lower by 13.9 kcal mol^−1^ than unsubstituted IM-A4. Computed Δ*G*^‡^ in different DFT functionals is consistent with very little variation (10–18 kcal mol^−1^). When –NMe_2_ is replaced by –NH_2_, Δ*G*^‡^ increases by approximately 4 kcal mol^−1^ which is obvious due to lack of its electron donation capability. On the other hand, difference in Δ*G*^‡^ between –OH and –OMe is very small (approximately 1.5 kcal mol^−1^) as calculated by different DFT functionals. Computed Δ*G*^‡^ has been found maximum for –NO_2_ which is (approximately 3 kcal mol^−1^) larger than unsubstituted IM-A4. By contrast, computed Δ*G*^‡^ for –COOH has been found little lowered which is obvious due to its weak –M effect.

**Figure 6.**
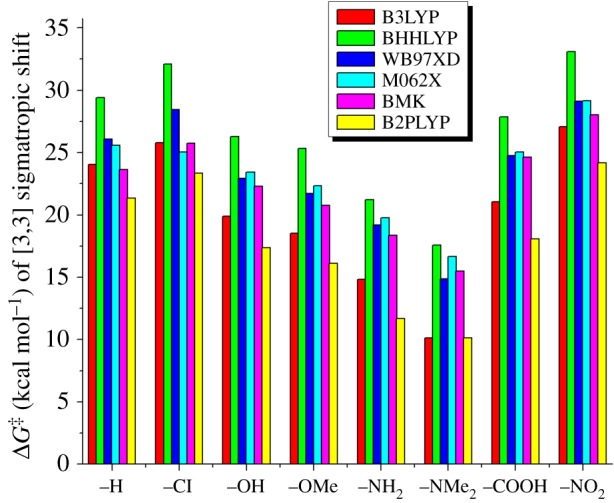
Comparative DFT performance of activation energy parameters for different substituents.

Figure 6 displays the comparative evolution of activation energy parameter in different DFT functionals. BHHLYP estimated Δ*G*^‡^ and Δ*H*^‡^ were larger, by a significant margin, than B3LYP. Although B2PLYP estimated Δ*G*^‡^ are little off suited, Δ*H*^‡^ is very consistent with B3LYP. On the other hand, Truhlar's M062X, Bose's ‘*τ*’ dependent BMK and Grimme's dispersion corrected *ω*B97XD give close estimates of Δ*G*^‡^ for present investigation, but Δ*H*^‡^ is widely varied. Thus, BHHLYP and B2PLYP do not prove to be reliable in predicting Δ*G*^‡^ parameter, but M062X, *ω*B97XD and BMK are equally acceptable with B3LYP.

## 4. Conclusion

The current computational study reveals the theoretical investigation of Banert cascade reaction with B3LYP/6-31G(d,p) level of theory. Two probable mechanistic paths were considered, which include direct displacement of chloride ion by azide anion (path A) and SN2-type attack at γ carbon atom of C≡C triple bond (path B). On the basis of our gas-phase calculation, path A is more favourable than path B for the Banert cascade reaction as it has low activation energy barrier of TS-A1 (3.94 kcal mol^−1^) when compared with TS-B1 in path B (5.1 kcal mol^−1^). Further upon solvation, activation energy barrier of TS-A1 stabilized, whereas activation energy of TS-B1 increased significantly. Nevertheless, path A is followed by key intermediate propargyl azide which was further converted into allenyl azide via [3,3] sigmatropic rearrangement. In addition to this functional group, substituent effects on [3,3] sigmatropic rearrangement of propargyl azide were also studied. It was observed that electron-donating group decreased the activation energy of sigmatropic rearrangement, whereas electron-withdrawing group increased activation energy barrier considerably. The most important part of this reaction is the formation of triazafulvene. Owing to its high dipole moment, triazafulvene can be easily attacked by any desired external nucleophile present in the reaction which can further lead to benzylic-substituted 1,2,3-triazoles to attach with any molecular architecture. We have demonstrated the influence of functional group for this reaction. This can further guide the experimentalist to design benzylic-substituted 1,2,3-triazoles which can be accessed via the Banert cascade reaction. The effects of different DFT functionals on activation energy of sigmatropic rearrangement were also calculated. While BHHLYP overestimates Δ*G*^‡^ and Δ*H*^‡^, M062X, *ω*B97XD and BMK predicted that Δ*G*^‡^ is rather consistent with B3LYP.

## Supplementary Material

supplementary materials for optimized structures

## Data accessibility

The datasets supporting this article have been uploaded as part of the electronic supplementary material.
