# Supplementary material for: Theoretical investigation of Banert cascade reaction
Source: R Soc Open Sci. 2018 Apr 4;5(4):171075. doi: 10.1098/rsos.171075 (PMC5936888; doi:10.1098/rsos.171075)

**Royal Society Open Science**

**Electronic Supplementary Material**

Theoretical Investigation of Banert-Cascade Reaction

Shubhankar Bhattacharyya^a^*, Kaushik Hatua^b^*

*^a^Chemistry of Interfaces, Luleå Tekiska Universitet, Luleå-97187, Sweden*

*^‡^Department of Chemistry, Indian Institute of Engineering Science and Technology, Shibpur-711103, India*

*Corresponding authors: shubhankar.bhattacharyya@ltu.se; kaushikhatua@gmail.com*

| **Table S1**: B3LYP/6-31G(d,p) calculated relative Gibb’s free energy (ΔG^‡^) and Enthaply (ΔH^‡^) of intermediates and transition states in gas phase and in different solution | | | | | | | | | | | | | | |
| --- | --- | --- | --- | --- | --- | --- | --- | --- | --- | --- | --- | --- | --- | --- |
|  | Gas | | Acetone | | Ethanol | | Methanol | | Acetonitrile | | DMSO | | Water | |
|  | ΔG^‡^ | ΔH^‡^ | ΔG^‡^ | ΔH^‡^ | ΔG^‡^ | ΔH^‡^ | ΔG^‡^ | ΔH^‡^ | ΔG^‡^ | ΔH^‡^ | ΔG^‡^ | ΔH^‡^ | ΔG^‡^ | ΔH^‡^ |
| IM-A1 | -5.55 | -14.66 | 9.36 | -0.52 | 9.30 | -0.41 | 9.66 | -0.28 | 9.71 | -0.24 | 9.82 | -0.16 | 9.98 | -0.04 |
| TS-A1 | -1.61 | -11.87 | 12.86 | 2.06 | -14.11 | 2.17 | 16.53 | 2.30 | 13.19 | 2.33 | 13.28 | 2.42 | 13.44 | 2.53 |
| IM-A2 | -19.72 | -27.91 | -13.66 | -23.96 | -13.83 | -23.95 | -13.58 | -23.93 | -13.57 | -23.92 | -13.54 | -23.91 | -13.50 | -23.88 |
| IM-A3 | -12.50 | -14.29 | -19.25 | -23.82 | -19.51 | -23.90 | -19.36 | -23.99 | -13.37 | -24.01 | -22.54 | -24.07 | -19.45 | -24.15 |
| IM-A4 | -12.51 | -14.17 | -10.12 | -23.11 | -21.29 | -23.19 | -21.14 | -23.28 | -21.16 | -23.31 | -21.20 | -23.36 | -21.24 | -23.45 |
| TS-A2 | 11.53 | 7.13 | 3.08 | -1.91 | 2.82 | -2.00 | 2.96 | -2.09 | 2.95 | -2.11 | 2.91 | -2.17 | 2.87 | -2.25 |
| IM-A5 | -17.06 | -20.64 | -24.19 | -28.41 | -24.44 | -28.48 | -24.28 | -28.55 | -24.29 | -28.57 | -24.32 | -28.63 | -24.35 | -28.70 |
| IM-A6 | -37.66 | -43.04 | -50.05 | -56.22 | -50.35 | -56.36 | -50.26 | -56.50 | 52.65 | -57.40 | -51.00 | -57.21 | -50.47 | -56.78 |
| TS-A3 | -36.46 | -43.20 | -48.94 | -56.47 | -49.25 | -56.61 | -49.16 | -56.76 | -50.92 | -57.10 | -49.27 | -56.90 | -49.37 | -57.04 |
| IM-A7 | -38.34 | -43.64 | -50.67 | -56.77 | -50.98 | -57.50 | -50.89 | -57.06 | -51.29 | -57.54 | -50.37 | -56.65 | -51.10 | -57.34 |
| IM-B1 | -5.67 | -14.66 | 9.43 | -0.52 | 9.36 | -0.40 | 9.73 | -0.27 | 9.77 | -0.24 | 9.88 | -0.15 | 10.03 | -0.03 |
| TS-B1 | -0.57 | -10.18 | 16.18 | 5.06 | 15.59 | 4.66 | 17.01 | 5.25 | 16.53 | 5.36 | 16.65 | 5.46 | 16.81 | 5.59 |
| IM-A5 | -17.06 | -20.64 | -24.19 | -28.41 | -24.44 | -28.48 | -24.28 | -28.55 | -24.29 | -28.57 | -24.32 | -28.63 | -24.35 | -28.70 |

**Cartesian coordinates of IM-A1**

C -0.4701640 -1.1072460 -0.3110050

C 0.5033210 -0.4067890 -0.1413480

C -1.6743200 -1.9182880 -0.4829880

H -1.3978260 -2.9813310 -0.5542630

H -2.1438290 -1.6524550 -1.4386270

C -2.6965170 -1.7043150 0.6485250

H -3.5963770 -2.3056460 0.4693960

H -2.2728590 -1.9983390 1.6148610

H -2.9585970 -0.6419590 0.6813970

C 1.6039580 0.4932630 0.0533450

H 1.7106290 1.2223350 -0.7440570

H 1.5746640 1.0070700 1.0092670

Cl 3.2915130 -0.4442440 0.0692380

N -0.3853090 2.5269790 0.1494020

N -1.4512180 2.0332230 -0.0369850

N -2.5162150 1.5487580 -0.2287290

**Cartesian coordinates of IM-A2**

C -1.9921710 0.0028680 -0.6132580

C -0.8079540 0.1253470 -0.8381440

C -3.4070400 -0.1812530 -0.2890470

H -3.8975480 -0.7467130 -1.0941710

H -3.9107750 0.7958070 -0.2637340

C -3.6334160 -0.9059090 1.0510070

H -4.7042940 -1.0281900 1.2512810

H -3.1656820 -1.8939790 1.0376060

H -3.1884320 -0.3407330 1.8746630

C 0.6207460 0.2734940 -1.1093240

H 0.8176740 0.1349150 -2.1759380

H 1.2337170 -0.4891010 -0.5651310

Cl 2.6441880 -2.0007210 0.2297810

N 1.1099320 1.6599560 -0.8152650

N 1.2909870 1.9389290 0.3703390

N 1.4823890 2.3572530 1.4194610

**Cartesian coordinates of IM-A3**

C -0.3120770 0.0459740 0.6736610

C 0.8746970 0.2798470 0.6404280

C -1.7504550 -0.2228680 0.7046870

H -2.1873870 0.2766450 1.5791810

H -1.9086800 -1.2976780 0.8616820

C -2.4831270 0.2286910 -0.5719620

H -3.5510650 0.0034160 -0.4960730

H -2.3687560 1.3050130 -0.7272260

H -2.0848870 -0.2841880 -1.4515830

C 2.3115680 0.5326640 0.5618970

H 2.5057970 1.4639890 0.0116170

H 2.7407750 0.6545600 1.5597580

N 3.1040020 -0.5749510 -0.0494380

N 2.6945540 -0.9739520 -1.1455510

N 2.4150440 -1.4371690 -2.1510750

**Cartesian coordinates of IM-A4**

C -0.1555630 0.6340430 0.0368580

N 2.2892810 0.6757420 -0.4022760

N 2.4600930 -0.4861650 -0.0153230

N 2.7239320 -1.5636780 0.2550460

C 1.0998440 1.3696330 0.1741320

H 1.0540810 2.3201240 -0.3636270

H 1.2930900 1.6054320 1.2300430

C -1.1720070 -0.0092090 -0.0944710

C -2.4201150 -0.7600140 -0.2402550

H -2.5265290 -1.0810260 -1.2844060

H -2.3597480 -1.6793420 0.3562340

C -3.6611790 0.0492090 0.1807580

H -3.7612380 0.9534500 -0.4257350

H -4.5658030 -0.5526720 0.0537100

H -3.5928760 0.3507630 1.2295250

**Cartesian coordinates of IM-A5**

C 0.2069850 1.0478660 -0.0000230

N -3.0895010 -0.1484450 -0.0000410

N -2.0206760 -0.5459670 0.0000440

N -0.9158240 -1.1048190 0.0000570

C 0.1376910 2.3526960 0.0000380

H 0.1086880 2.9269890 0.9254610

H 0.1085180 2.9270560 -0.9253380

C 0.2543700 -0.2665960 -0.0000850

C 1.5174440 -1.0975780 0.0000110

H 1.4810860 -1.7600230 0.8748270

H 1.4811000 -1.7601400 -0.8747130

C 2.8116430 -0.2859770 -0.0000300

H 2.8781400 0.3562550 0.8831180

H 3.6775170 -0.9541020 0.0000300

H 2.8781580 0.3561230 -0.8832730

**Cartesian coordinates of IM-A6**

C -0.7759400 0.6230250 -0.0768860

C -0.8513330 1.9165860 -0.4158230

H 0.0042290 2.5785940 -0.3310350

H -1.7847520 2.3303630 -0.7823100

C 0.3275030 -0.1915580 0.4505350

C 1.7412240 0.2347620 0.6997730

H 2.1836750 -0.4682950 1.4104610

H 1.7473290 1.2249410 1.1716920

C 2.5825500 0.2696440 -0.5910280

H 3.6069380 0.5802260 -0.3665040

H 2.6177000 -0.7204890 -1.0532720

H 2.1688740 0.9697180 -1.3235540

N -0.1058020 -1.3940130 0.6464400

N -1.5134740 -1.3954840 0.2449310

N -1.8852330 -0.2642280 -0.1609940

**Cartesian coordinates of IM-A7**

C -0.9304060 0.7028920 0.1010450

C -1.2552640 1.9511800 0.4603130

H -0.4980770 2.7047300 0.6508210

H -2.2971640 2.2333020 0.5678220

C 0.3678340 0.0490690 -0.1192960

C 1.7234990 0.6715660 0.0208990

H 1.8199000 1.0563560 1.0458910

H 1.7633650 1.5582830 -0.6270320

C 2.8823220 -0.2744530 -0.3015600

H 3.8379610 0.2433950 -0.1803950

H 2.8713320 -1.1451560 0.3583780

H 2.8125290 -0.6419190 -1.3281760

N 0.1577600 -1.1793880 -0.4608140

N -1.2967330 -1.3613680 -0.4720920

N -1.9016230 -0.3054280 -0.1568280

**Cartesian coordinates of IM-B1**

C 0.4896210 -0.3964730 -0.1734290

C -0.4981080 -1.0746020 -0.3512760

C 1.6075600 0.4783440 0.0372930

H 1.7077910 1.2415610 -0.7283850

H 1.6065050 0.9482800 1.0159880

N -2.5019650 1.6012900 -0.1027380

N -1.4258570 2.0621160 0.0852650

N -0.3489420 2.5326360 0.2684160

C -1.7182580 -1.8594730 -0.5316830

H -2.2063570 -1.5416010 -1.4617420

H -1.4599340 -2.9217450 -0.6598560

C -2.7097680 -1.6849770 0.6334270

H -2.2676790 -2.0300690 1.5742810

H -2.9549880 -0.6218180 0.7221240

H -3.6226410 -2.2645460 0.4485090

Cl 3.2808800 -0.4822270 -0.0267040

**Cartesian coordinates of IM-B2**

C 0.0136020 -0.0034410 0.8018910

C 1.2413010 -0.3044270 0.4296480

C -1.2146700 0.2620230 1.1490930

H -1.4353400 0.5966590 2.1646660

H -2.1019510 0.1438900 0.4813560

N 2.1757720 0.6488160 -0.1402430

N 1.8092340 1.8141790 -0.2975400

N 1.5903430 2.9230310 -0.4823100

C 1.8550300 -1.6825970 0.5178490

H 2.8013270 -1.6198110 1.0745760

H 1.1781810 -2.3147220 1.0994620

C 2.1173380 -2.3187800 -0.8559440

H 1.1801360 -2.4434370 -1.4067740

H 2.7797510 -1.6866900 -1.4554580

H 2.5886740 -3.3029980 -0.7501370

Cl -4.1231660 -0.1642210 -0.4130740

**Cartesian coordinates of IM-B3**

C -0.2095330 1.3463330 0.0883290

C 0.3641230 0.2090070 -0.2398750

C -0.7848380 2.4753630 0.4063770

H -1.1042440 3.1906670 -0.3507870

H -0.9691300 2.7543610 1.4432280

N -0.3580870 -1.0105100 -0.4856350

N -1.5894960 -0.9834550 -0.3607050

N -2.7227030 -1.0817210 -0.2789220

C 1.8559050 0.0197390 -0.3929310

H 2.0487720 -0.3716480 -1.4001120

H 2.3339980 1.0011000 -0.3302400

C 2.4525780 -0.9351000 0.6516240

H 2.3138860 -0.5433180 1.6640790

H 1.9756380 -1.9176810 0.5994610

H 3.5254710 -1.0674710 0.4808650

**Cartesian coordinates of TS-A1**

C -0.5376700 -1.0436370 -0.2341880

C 0.3948640 -0.2841540 -0.0539820

C -1.6230240 -2.0042480 -0.4099230

H -1.2296030 -3.0234200 -0.2804790

H -1.9920310 -1.9460420 -1.4423990

C -2.7943230 -1.7658160 0.5603960

H -3.6022050 -2.4831190 0.3735620

H -2.4653840 -1.8797150 1.5980620

H -3.1715570 -0.7487070 0.4299200

C 1.3346410 0.7418700 0.1155500

H 1.8651340 1.1515070 -0.7262710

H 1.5055140 1.1814760 1.0826690

Cl 3.4012220 -0.5302180 0.5470230

N -0.1105640 2.4375730 -0.1937150

N -1.1911610 1.9761790 -0.4208810

N -2.2422040 1.5034750 -0.6448020

**Cartesian coordinates of TS-A2**

C 0.1533970 1.0276140 -0.0000260

N 2.5385280 -0.0896990 -0.0001320

N 1.7244710 -0.9586230 -0.0000480

N 0.6628700 -1.4948330 -0.0000610

C 1.4210140 1.5543310 0.0001020

H 1.8142230 1.9926530 -0.9143780

H 1.8141210 1.9923390 0.9147840

C -0.5945990 0.0200370 0.0001270

C -1.9237240 -0.6174720 0.0002350

H -1.9947180 -1.2744370 -0.8746070

H -1.9948770 -1.2738350 0.8755200

C -3.0652090 0.4100730 -0.0002130

H -3.0168610 1.0502550 -0.8853960

H -4.0311540 -0.1031910 -0.0001710

H -3.0170820 1.0508020 0.8845880

**Cartesian coordinates of TS-A3**

C -0.9550090 0.6356360 -0.0106850

C -1.1750550 1.9549620 -0.0812550

H -0.3792060 2.6750630 0.0783330

H -2.1685000 2.3303540 -0.3022770

C 0.2603520 -0.1414840 0.2628420

C 1.6422950 0.3765410 0.5343240

H 1.9570000 0.0075940 1.5172230

H 1.6224330 1.4698460 0.6009400

C 2.6596240 -0.0732020 -0.5285510

H 3.6597750 0.2914980 -0.2773720

H 2.6885850 -1.1636150 -0.5868750

H 2.3944630 0.3122520 -1.5177540

N -0.0376670 -1.3994530 0.2178370

N -1.4656370 -1.4741790 -0.0860120

**Cartesian coordinates of TS-B1**

C 0.0180330 1.5187520 0.0887030

C 0.7776050 0.5780480 -0.1990380

C -1.0047770 2.3769280 0.3732330

H -1.6133950 2.7765600 -0.4260480

H -1.4537320 2.3757440 1.3567300

N -0.4094430 -1.2118610 -0.5075230

N -1.4863490 -0.7363590 -0.2927160

N -2.4821720 -0.1610220 -0.0646310

C 2.0971210 -0.0318440 -0.4359400

H 2.0728590 -0.5781310 -1.3857270

H 2.8272790 0.7822100 -0.5469390

C 2.5382830 -0.9843470 0.6863380

H 2.6192820 -0.4500650 1.6384150

H 1.7994690 -1.7805380 0.8002630

H 3.5136920 -1.4301340 0.4572000

Cl -0.2278720 4.6107530 0.8071340

**B3LYP/6-31G* calculated IR spectra of different intermediates and transition states**


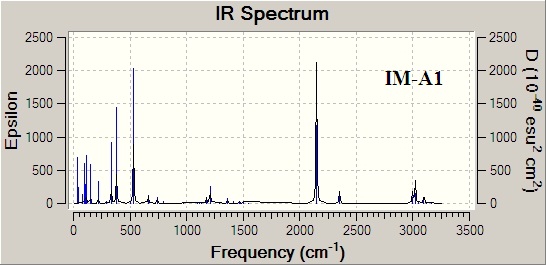


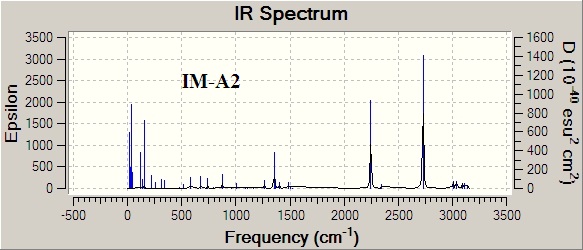


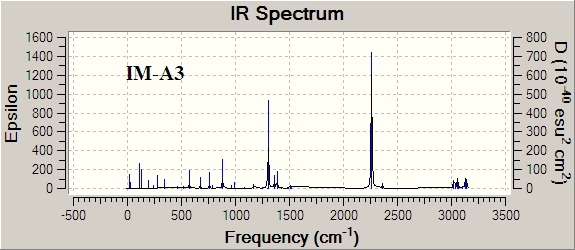


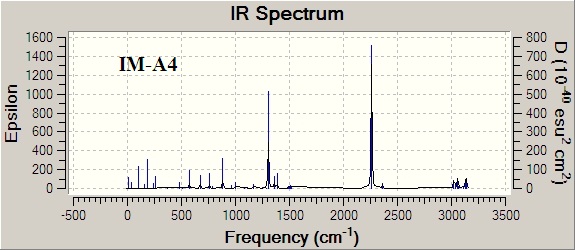


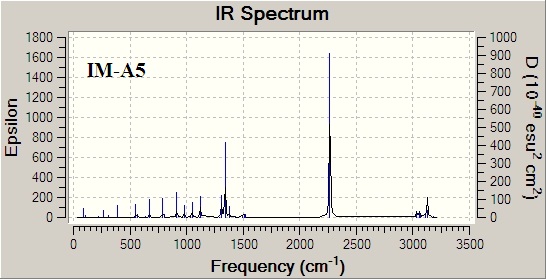


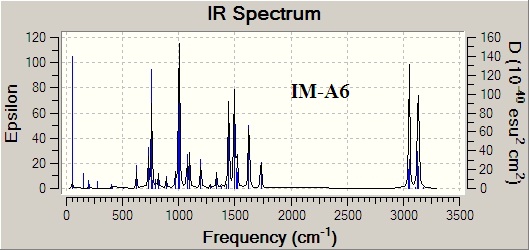


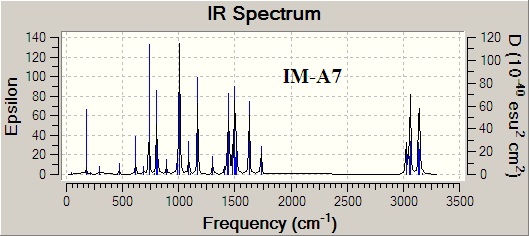


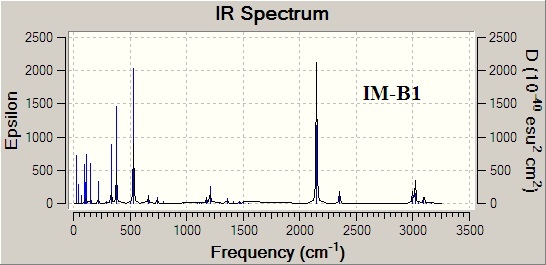


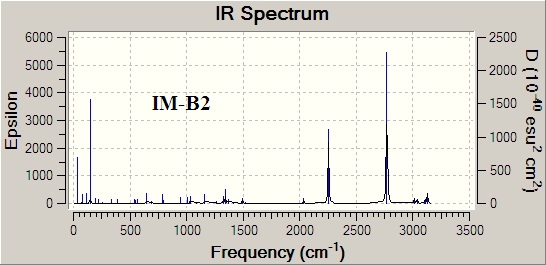


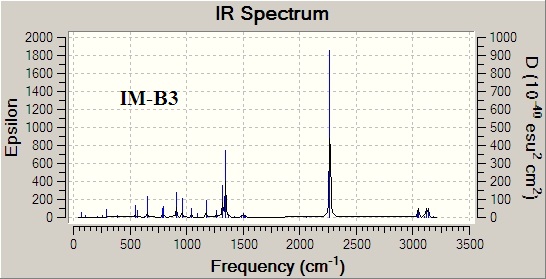


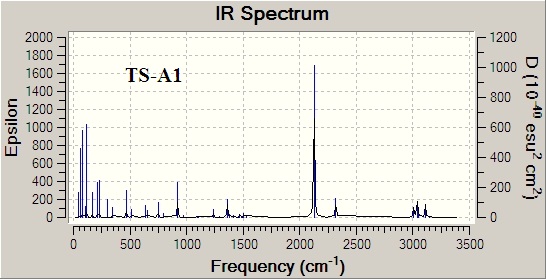


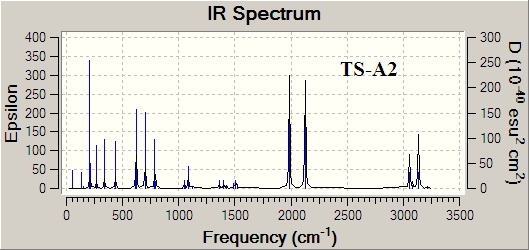


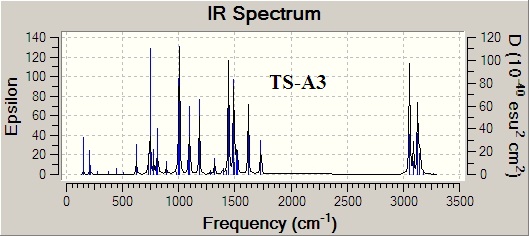


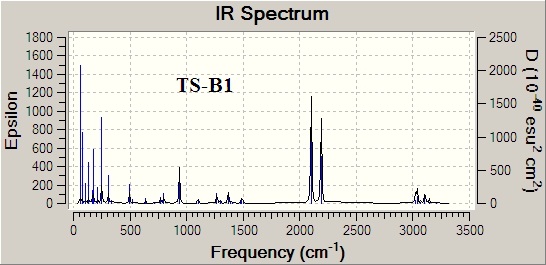

Supplement: supplementary materials for optimized structures [file rsos171075supp1.docx]
